# Subclavian-to-descending aortic bypass for the treatment of severe late-stage aortic coarctation in a 62-year-old adult: a case report and literature review

**DOI:** 10.3389/fcvm.2025.1635499

**Published:** 2025-10-09

**Authors:** Bowen Wang, Zhan Zhang, Ke Chen, Xing Zhou, Fentang Gao, Ping Xie

**Affiliations:** ^1^ Department of Cardiology, Gansu Provincial Hospital, Lanzhou, Gansu, China; ^2^ Department of Integrated Chinese and Western Medicine, Gansu University of Traditional Chinese Medicine, Lanzhou, Gansu, China

**Keywords:** aortic coarctation, hypertension, vascular surgical procedures, collateral circulation, multidisciplinary care

## Abstract

**Background:**

Aortic coarctation (CoA) is a congenital cardiovascular condition usually diagnosed in infancy or childhood. Cases in adults are rare and often go undetected because symptoms can be obscured by extensive collateral circulation.

**Case description:**

A 62-year-old male was admitted to Gansu Provincial Hospital in December 2024 with complaints of recurrent chest tightness, shortness of breath, blurred vision, and tinnitus persisting for over two months. Physical examination revealed significant blood pressure discrepancies between the upper and lower extremities (>50 mmHg). Imaging confirmed severe CoA with nearly complete interruption of the descending aorta, extensive collateral circulation, and complications, including hypertensive crisis with cerebral hemorrhage likely due to extreme hypertension, and bronchiectasis with active pulmonary infection. After multidisciplinary team evaluation, left subclavian artery-to-descending aorta bypass grafting was performed. Postoperatively, blood pressure normalized across all limbs, and the patient remained asymptomatic at the six-month follow-up, with patent graft flow.

**Conclusion:**

This case of severe CoA in a 62-year-old male highlights the importance of early recognition of atypical presentations in adult patients, the need for individualized surgical strategies, and the benefits of long-term follow-up to ensure successful management and optimal outcomes.

## Introduction

Aortic coarctation (CoA) is a congenital cardiovascular anomaly characterized by a localized narrowing of the aortic lumen, most commonly at the isthmus near the ductus arteriosus (1). It accounts for approximately 5%–8% of all congenital heart defects, with an incidence of 3–4 per 10,000 live births (2). CoA is typically diagnosed in infancy or early childhood; however, in rare cases, it may remain undetected until adulthood due to compensatory development of extensive collateral circulation. In adults, the presence of well-established collaterals can obscure classical clinical signs—such as significant discrepancies in blood pressure between the upper and lower extremities—leading to delayed diagnosis (3). When left untreated, longstanding CoA can result in serious complications, including uncontrolled hypertension, intracranial hemorrhage, aortic rupture, and end-organ damage (4). This case report presents a rare instance of severe, undiagnosed CoA in a 62-year-old male, complicated by hypertensive crisis, cerebral hemorrhage, and coexisting bronchiectasis. The case highlights the diagnostic challenges posed by atypical adult presentations and underscores the importance of multidisciplinary collaboration in formulating individualized surgical strategies.

## Case description

A 62-year-old male was admitted to Gansu Provincial Hospital in April 2024 with a two-month history of recurrent chest tightness and shortness of breath. These symptoms were accompanied by bilateral blurred vision and high-frequency tinnitus. The patient denied vertigo, syncope, or visual blackout. His family history was unremarkable, and he had no known hereditary conditions. It is noteworthy that, although the patient had longstanding left ventricular hypertrophy, he had not previously experienced classic symptoms of coarctation. This was primarily due to well-developed collateral circulation—including markedly dilated internal thoracic and intercostal arteries—which effectively reduced the pressure gradient and maintained distal perfusion, thereby masking clinical manifestations for many years. Retrospectively, the patient did report a progressive decline in exercise tolerance in the two months prior to admission, but these symptoms became prominent only after the onset of a pulmonary infection (bronchiectasis with active infection). The infection likely increased metabolic demand, exceeded the collateral circulation's compensatory capacity, and triggered overt cardiopulmonary and neurological symptoms, as well as exacerbated the hypertensive crisis.

Two months prior to admission, following a common cold, the patient developed persistent chest discomfort, exertional dyspnea, productive cough with yellow sputum, and progressive reduction in exercise tolerance. Neurological symptoms, including blurred vision and tinnitus, developed concurrently. At the Second Hospital of Lanzhou University, the primary focus was on the neurological symptoms, and no systematic cardiovascular physical examination was performed; only cranial computed tomography (CT) was reported. Cranial CT revealed a cerebral hemorrhage. During the same hospitalization, chest CT incidentally identified features suggestive of CoA, marking the first recognition of this congenital anomaly.

Upon admission to our institution, significant interlimb blood pressure discrepancies were noted: 163/91 mmHg (right upper limb), 159/88 mmHg (left upper limb), 110/82 mmHg (right lower limb), and 102/69 mmHg (left lower limb). On physical examination, cardiac auscultation did not reveal any characteristic murmur suggestive of aortic coarctation (CoA). Cranial CT confirmed a hemorrhagic lesion involving the left basal ganglia, thalamus, and posterior lateral ventricle (approximately 9 ml), alongside multiple chronic infarcts, leukoencephalopathy, and cerebral atrophy. Chest CT revealed multiple bronchiectases with infection in the left lung with concurrent infection, as well as a severe “sandglass-shaped” narrowing at the aortic isthmus, with a minimum lumen diameter of 5 mm and calcification of the descending aortic wall. The aortic sinus was aneurysmally dilated to approximately 46 mm.

**Figure 1:**
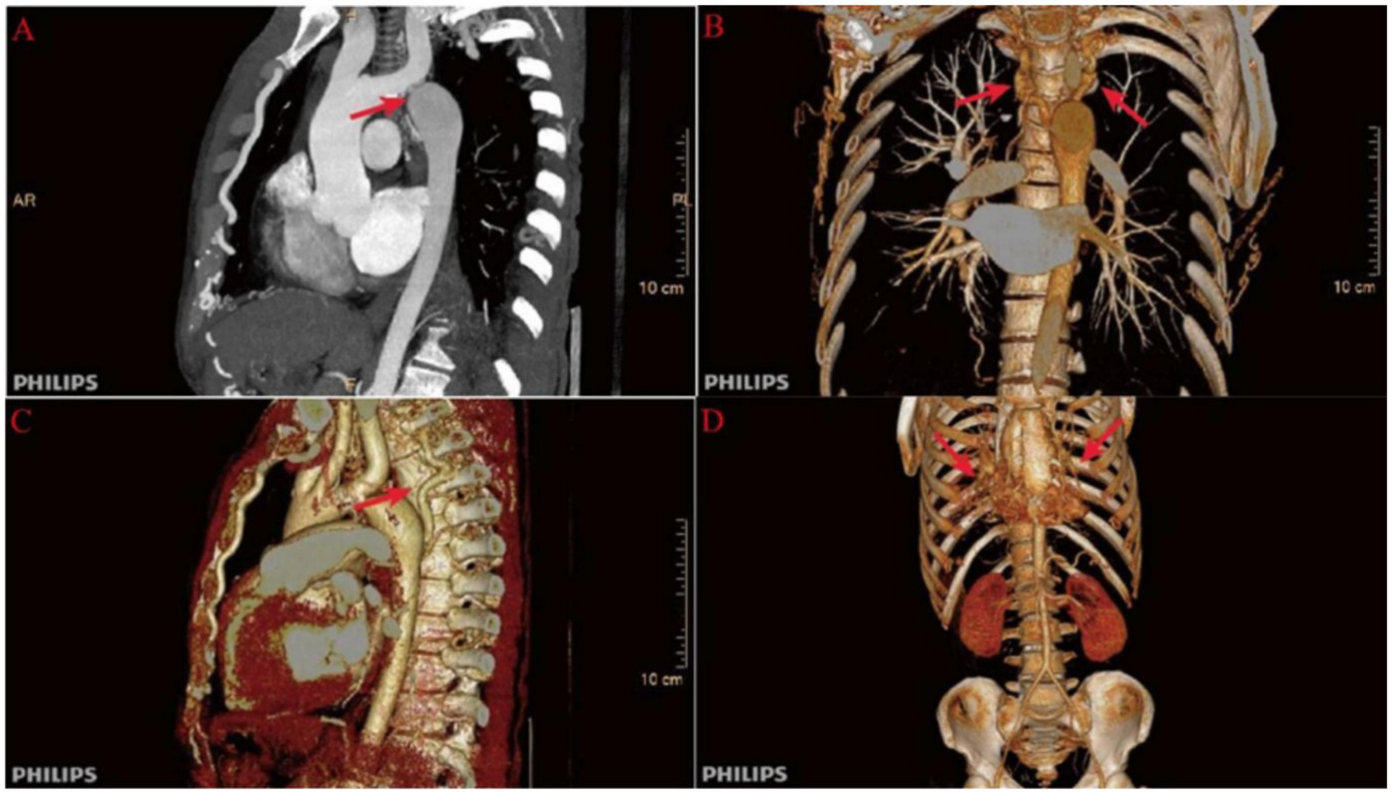
Preoperative computed tomography angiography (CTA) findings. **(A)** Severe narrowing at the aortic isthmus (“sandglass-shaped” coarctation, arrow); **(B, C)** Markedly dilated and tortuous paravertebral arteries, demonstrating extensive collateral circulation; **(D)** Prominent internal thoracic arteries serving as major collateral pathways.

Further evaluation using computed tomography angiography (CTA) confirmed severe CoA, with near-complete interruption of the descending aorta at the isthmus and extensive collateral circulation, including prominently dilated and tortuous internal thoracic arteries (Figure 1). The renal arteries were assessed and found to be unremarkable, with preserved renal function throughout hospitalization.

Transthoracic echocardiography revealed a dilated aortic sinus, significant left ventricular hypertrophy, and mild mitral regurgitation, with preserved left ventricular systolic function. The aortic valve was tricuspid. The ascending aorta was within normal limits, while the aortic arch and descending aorta were poorly visualized. A measurable pressure gradient across the coarctation was not obtained by echocardiography due to poor acoustic windows and the complex anatomy. Abdominal and upper limb vascular ultrasound showed a hyperechoic hepatic lesion consistent with hemangioma, along with bilateral carotid intima-media thickening and atherosclerotic plaques. No abnormalities were found in the upper limb arteries or veins.

Cranial magnetic resonance imaging (MRI) showed multiple old lacunar infarcts, gliosis, demyelination (Fazekas grade I), and senile cerebral changes. Magnetic resonance angiography (MRA) revealed no significant abnormalities. Laboratory tests were unremarkable. Based on these findings, the patient was diagnosed with: (1) aortic coarctation complicated by hypertensive crisis, (2) cerebral hemorrhage (recovery phase), and (3) bronchiectasis with active infection.

Initial treatment included triple antihypertensive therapy with nifedipine controlled-release tablets (30 mg once daily), metoprolol sustained-release tablets (47.5 mg once daily), and sacubitril/valsartan (200 mg twice daily), combined with anti-infection therapy. In this case, the use of sacubitril/valsartan was an individualized clinical decision based on the patient's persistent hypertension despite long-term calcium channel blocker therapy and potential cardiac remodeling factors. Although not a first-line antihypertensive option, this regimen partially alleviated the patient's symptoms given the complex clinical context. It must be emphasized that strict evaluation of indications is mandatory when considering its use in patients without left ventricular dysfunction.

**Figure 2:**
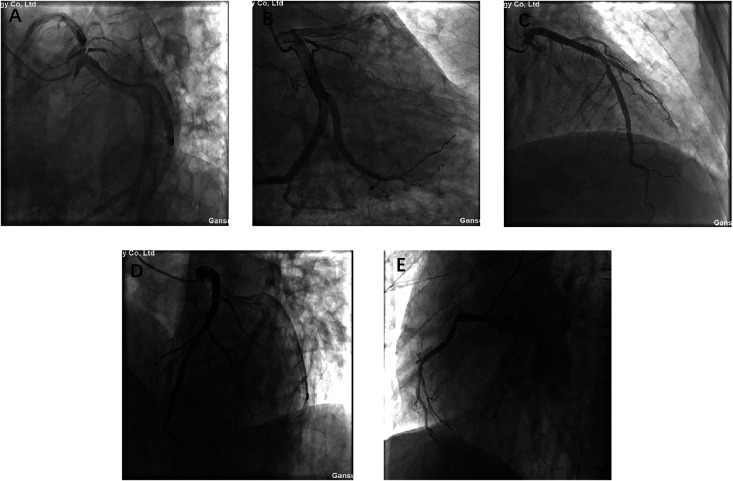
**A**, **B**, **C**, **D**, and **E** present coronary angiograms in different projections. The results indicate: dilation of the left main coronary artery, plaque in the mid-to-distal segment of the anterior descending branch, diffuse dilation of the circumflex artery, hypoplasia of the right coronary artery, no significant coronary stenosis, and normal blood flow.

Prior to surgery, coronary angiography was performed due to the patient's age and risk factors. The angiogram demonstrated left main trunk dilation, mild plaques in the mid-left anterior descending artery, diffuse dilation of the circumflex artery, and a diminutive right coronary artery, but no significant coronary artery stenosis (Figure 2). These findings effectively excluded significant coronary artery disease preoperatively.

Regarding surgical planning, traditional resection of the coarctation with replacement via left-sided thoracotomy was considered. However, this approach was deemed technically unfeasible and high risk due to (1) the nearly complete aortic interruption and severe calcification evident on imaging, (2) extensive collateral circulation, (3) the risk of complications from aortic arch clamping (including potential spinal ischemia due to interruption of collateral vertebral flow), (4) the patient's comorbidities (recent cerebral hemorrhage and active pulmonary infection), which increased the risks of cardiopulmonary bypass and postoperative complications. Therefore, an extra-anatomic bypass from the left subclavian artery to the descending aorta was selected as the optimal approach, as it avoided direct manipulation of the aortic arch and did not require cardiopulmonary bypass. This strategy is supported by recent literature, which indicates that extra-anatomic bypass provides favorable outcomes in elderly or complex adult CoA cases (7).

After comprehensive evaluation and multidisciplinary team (MDT) discussion, surgical correction was deemed necessary. Given the patient's age, vascular anatomy, and the extent of aortic interruption with heavy calcification, endovascular intervention was considered unfeasible. An extra-anatomic bypass procedure using a left subclavian artery-to-descending aorta synthetic graft was selected as the optimal approach. The left subclavian artery, with an internal diameter of 15 mm, was suitable as the proximal anastomosis site. A post-stenotic aneurysm of the descending aorta was also noted, measuring approximately 54.0 mm × 33.2 mm in maximal cross-sectional diameter.

## Surgical procedure details

After induction of general anesthesia and endotracheal intubation, right upper limb blood pressure was 182/96 mmHg, and right lower limb blood pressure was 106/75 mmHg. A standard left thoracotomy was performed through the fourth intercostal space, encountering dense pleural adhesions and abundant collateral vessels (ligated using 7-0 sutures and electrocautery). Single-lung ventilation was initiated to facilitate exposure. The left subclavian artery (proximal segment) and descending aorta (distal to the coarctation) were carefully dissected and isolated. The descending aorta was partially clamped, and a longitudinal arteriotomy (∼4 cm) was made. A 22 mm synthetic vascular graft (Dacron) was anastomosed end-to-side to the descending aorta using 4-0 Prolene, then reinforced with pledgeted sutures. The graft was clamped, and the aortic clamp released to assess for bleeding. The left subclavian artery was similarly clamped, incised, and anastomosed end-to-side to the other end of the graft. The graft was de-aired, and hemostasis confirmed. Intraoperative monitoring showed immediate improvement in lower limb blood pressure (right upper limb: 106/70 mmHg, right lower limb: 108/75 mmHg). No intraoperative complications occurred.

No postoperative anticoagulation or antiplatelet therapy was administered, in accordance with our institutional protocol for this procedure in the absence of other indications.

**Figure 3:**
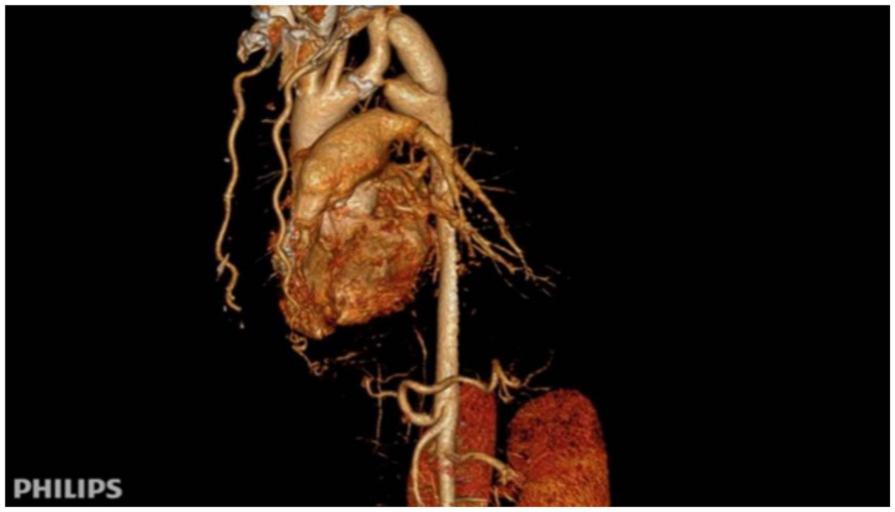
Immediate postoperative CTA following subclavian-to-descending aorta bypass. Patency of the synthetic vascular graft (Dacron) from the left subclavian artery to the descending aorta is demonstrated, with normalization of the aortic lumen distal to the coarctation.

After surgery, the patient's antihypertensive regimen was gradually reduced in a stepwise fashion. At discharge, only metoprolol was continued, and both nifedipine and sacubitril/valsartan were discontinued. Postoperative blood pressure measurements were balanced across all four limbs: 115/69 mmHg (right upper limb) and 126/75 mmHg (left lower limb). Follow-up CTA confirmed satisfactory graft patency and flow (Figure 3).

**Figure 4:**
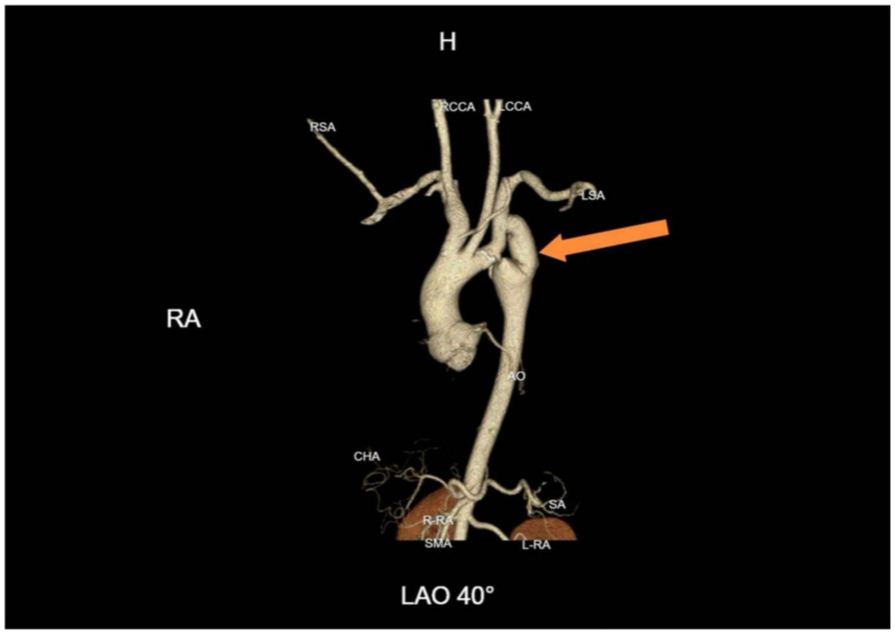
One-year follow-up CTA demonstrating graft patency. Continued patency of the bypass graft with no evidence of pseudoaneurysm, graft occlusion, or aneurysmal progression at the coarctation site.

**Figure 5:**
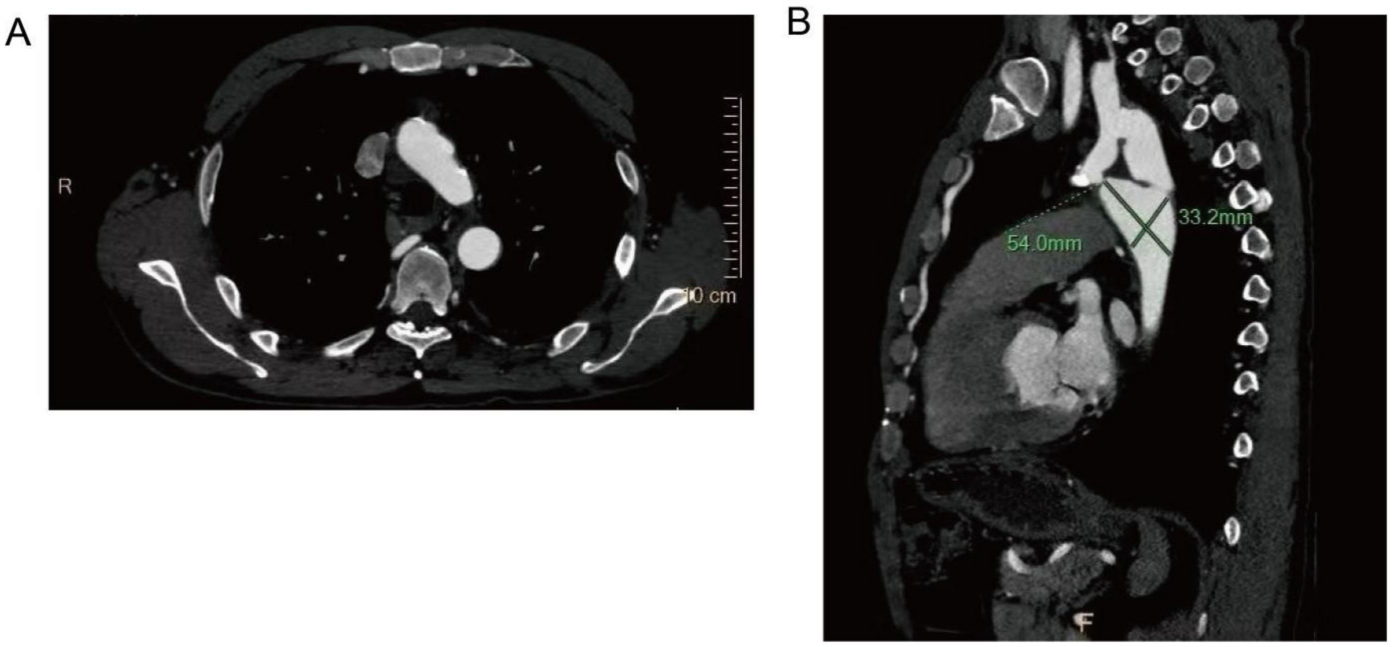
Scattered calcified plaques are observed in the walls of the aortic arch and brachiocephalic trunk, with irregular lumens as shown in **(A)**. The descending aorta shows poststenotic aneurysmal dilation, with dimensional measurements provided in **(B)**.

After more than six months of follow-up observation, the patient reported complete resolution of cardiopulmonary and neurological symptoms. Blood pressure remained well controlled, and there was no recurrence of symptoms. Serial imaging demonstrated continued graft patency without evidence of complications such as graft occlusion, pseudoaneurysm, infection, or aneurysmal progression (Figure 4). Additional axial CTA images are provided (Figure 5) to better illustrate the extent of aortic calcification.

To contextualize this case, we conducted a literature review of 21 previously reported cases of aortic coarctation in adults and elderly patients (Table 1). Patients ranged in age from 3 months to 65 years, though the majority were adults over 40 years old. Of these, seven underwent prosthetic graft replacement (with three requiring cardiopulmonary bypass), ten were treated with interventional (endovascular) therapy, and one was managed conservatively due to multi-organ involvement. Three cases—including the present patient—were treated with a left subclavian artery-to-descending aorta bypass, all of which had favorable outcomes and symptomatic improvement. The remainder were managed with either resection and prosthetic graft replacement or endovascular intervention (5, 6).

**Table 1:** Overview of published cases of aortic coarctation treatment.

No.	First author (Year)	Country	Age (years)	Sex	Diagnosis	Combined lesions	Treatment plan	Cardiopulmonary bypass	Postoperative complications	Follow-up	Main conclusion
(12)	Niwa K, et al. (2023)	Japan	56	M	CoA	–	Descending aorta graft replacement	Not mentioned	None	Not mentioned	Symptom improvement
(13)	Nicole Schulick, et al. (2022)	USA	3 months	F	CoA	–	LSA–DA–descending aortic bypass	None	None	7 months	Symptom improvement
(14)	Arun Sharma, et al. (2024)	India	24	M	CoA + BAV	3)	Ascending–DA–descending aortic bypass	Yes	None	6 months	Symptom improvement
(15)	D B H Verheijen, et al. (2022)	Netherlands	40	M	CoA	–	Stent implantation	None	None	20 months	Symptom improvement
(16)	Herrera Mingorance JD, et al. (2022)	Spain	41	M	CoA + LV dysfunction	–	Balloon angioplasty + stent	None	None	8 months	Symptom improvement
(17)	Hanazuka T, et al. (2022)	Japan	22	F	CoA	–	Balloon angioplasty + stent	Not mentioned	Re-stenosis after 2 years, hematuria	3 years	Prognosis unclear
(18)	Abuji K, et al. (2023)	India	16	F	CoA	3)	LSA–DA–descending aortic bypass	Not mentioned	None	1 year	Good recovery
(19)	Thanigai Arasu, et al. (2024)	India	6	M	CoA	–	Ascending–DA–descending aortic bypass	Yes	Recurrent leg weakness	Not mentioned	Symptom improvement
(20)	An M Van Berendoncks, et al. (2024)	Belgium	20	F	CoA	–	Stent implantation	None	None	2 years	Symptom improvement
(21)	Frank Molina-Ricaurte, et al. (2022)	Chile	41	M	CoA + LV dysfunction	–	Aortic graft + ascending–DA–descending bypass	Yes	None	30 months	Symptom improvement
(22)	Mitsuru Sato, et al. (2024)	Japan	6	F	CoA	–	Modified graft descending aortic replacement	None	None	8 years	Symptom improvement
(23)	Sawaka Tanabe, et al. (2022)	Japan	57	F	CoA + LV dysfunction	4)	LSA–DA–descending aortic bypass	Not mentioned	None	Not mentioned	Symptom improvement
(24)	Sanjay Tyagi, et al. (2022)	India	43	F	CoA	–	Balloon angioplasty + stent	Not mentioned	Mild leg weakness	2 years	Symptom improvement
(25)	Davide Reis, et al. (2022)	Portugal	23	M	CoA	–	Left cervical–DA bypass + stent	Not mentioned	None	Not mentioned	Symptom improvement
(26)	Zhang, et al. (2018)	China	40	M	CoA	3), 4), 5)	Conservative treatment	No	None	3 months	Good recovery
(27)	Onohara, et al. (2014)	Japan	65	M	CoA	3)	Aortic graft replacement	Yes	Not mentioned	6 months	Good recovery
(28)	Natraj Setty, et al. (2019)	India	9	M	CoA	4), 5)	Balloon angioplasty	No	Not mentioned	Not mentioned	Good recovery
(29)	Luo, et al. (2020)	China	44	M	CoA	4)	Balloon angioplasty + stent	Not mentioned	None	2 years	Good recovery
(30)	Ghaderian, et al. (2019)	Iran	3 months	M	CoA	5)	Balloon angioplasty + stent	Not mentioned	None	Not mentioned	Symptom improvement
(31)	Bayar, et al. (2015)	Turkey	41	M	CoA	1), 6)	Drug therapy	Not mentioned	Not mentioned	Not mentioned	Not mentioned
(32)	Armistead, et al. (2018)	USA	26	M	CoA	2), 6)	No surgical intervention	Not mentioned	Not mentioned	Not mentioned	Not mentioned

CoA, coarctation of the aorta; BAV, bicuspid aortic valve; LV, left ventricular; LSA, left subclavian artery; DA, descending aorta; –, not applicable/no combined lesion; 1) hypertension; 2) Turner syndrome; 3) bicuspid aortic valve; 4) left ventricular hypertrophy/dysfunction; 5) aortic valve regurgitation; 6) other congenital anomalies.

## Discussion

This case report presents a rare instance of CoA in a 62-year-old male, complicated by hypertensive crisis, cerebral hemorrhage, and bronchiectasis. Advanced imaging revealed a near-complete interruption of the descending aorta, accompanied by extensive collateral circulation. An extra-anatomic bypass graft from the left subclavian artery to the descending aorta was successfully performed, effectively eliminating the pressure gradient, normalizing systemic blood pressure, and relieving associated symptoms. At the six-months follow-up, the graft remained patent with no postoperative complications, highlighting the feasibility and durability of this surgical approach in the management of complex adult CoA.

Imaging modalities played a pivotal role in both the diagnosis and the management plan for this patient. In adults suspected of having CoA, contrast-enhanced CTA is generally considered the gold standard for anatomical assessment, allowing for detailed visualization of the coarctation site, extent of collateral formation, and degree of vascular calcification. Echocardiography offers valuable functional information, while magnetic resonance angiography provides a non-radiative alternative for follow-up and comprehensive vessel evaluation. Notably, given the history of cerebral hemorrhage, the patient underwent preoperative cerebrovascular assessment using cranial magnetic resonance angiography (MRA). The MRA results ruled out the presence of intracranial aneurysms, further supporting that the cerebral hemorrhage was more likely attributable to long-term hypertensive cerebrovascular injury rather than an inherent cerebrovascular malformation. This highlights the importance of comprehensive neurovascular imaging in guiding etiological assessment and treatment decisions for such patients with late-stage aortic coarctation presenting with neurological symptoms. In this case, CTA was critical not only in confirming the diagnosis but also in preoperative planning, particularly in assessing the suitability for different intervention options and mapping collateral circulation.

The review included 21 articles reporting 21 cases, with patient ages ranging from 3 months to 65 years. Seven underwent prosthetic graft replacement (three with cardiopulmonary bypass), ten received interventional therapy, and one was managed conservatively due to multi-organ involvement.

In this case, the collateral vessels, which included dilated internal thoracic, intercostal, and vertebral arteries, effectively reduced the pressure gradient across the stenotic segment, delaying end-organ damage and allowing prolonged survival. However, collateral circulation also obscured classical symptoms, leading to incidental detection during an unrelated examination. A similar phenomenon has been reported in previous studies, where the diagnosis of CoA in adults was often incidental and associated with complications such as intracranial hemorrhage, hypertensive crisis, or heart failure (7). This case highlights the need for heightened clinical vigilance in patients with unexplained hypertension, cardiac workload elevation, or neurological symptoms, particularly when associated with upper and lower limb blood pressure discrepancies.

Regarding the pathophysiological relationship between bronchiectasis and CoA in this patient, we propose that long-standing severe CoA resulted in marked dilation of collateral vessels (especially bronchial arteries), which may exert extrinsic compressive effects on adjacent bronchi. Chronic hypoperfusion distal to the coarctation may also contribute to airway ischemia and impaired mucociliary clearance, predisposing to recurrent infections and secondary bronchiectasis.

Given the rarity of adult CoA, especially with near-complete aortic interruption, there are few formal recommendations or consensus guidelines for its management, and most treatment strategies are based on case reports and small case series. The current literature suggests that endovascular stenting has become the first-line treatment for most adults with simple, discrete CoA due to its minimally invasive nature and favorable short-term outcomes (8). Importantly, the presence of aortic wall calcification is not considered an absolute contraindication for catheter-based intervention, and successful stenting has been reported even in calcified segments. However, in cases of severe anatomical abnormality—such as long-segment interruption, extreme tortuosity, or heavy calcification—endovascular approaches may not be feasible or safe, necessitating surgical solutions.

Traditional open repair methods, such as resection with end-to-end anastomosis or patch augmentation, are effective for certain CoA cases but may not be suitable for elderly patients or those with significant collateral circulation and fibrosis at the coarctation site (9). In this context, the left subclavian artery-to-descending aorta bypass grafting performed in this case proved to be a safe and effective alternative. This approach avoided the need for cardiopulmonary bypass, reduced intraoperative risks, and provided physiological hemodynamic improvement through an extra-anatomic bypass. The successful outcome in this patient demonstrates the feasibility and advantages of this technique in elderly patients with complex vascular anatomy.

The diagnostic methods used in this case, particularly CTA, were crucial in confirming the diagnosis and planning the surgical approach. CTA provided detailed visualization of the coarctation site, collateral circulation, and vascular calcification, which guided the choice of bypass grafting (7). Echocardiography and cranial imaging further supported the comprehensive assessment of the patient's condition. This case suggests that in hypertensive patients with persistent symptoms—such as blood pressure discrepancies, neurological manifestations (e.g., blurred vision or tinnitus), or refractory hypertension—vascular anomalies like CoA should be considered. Incorporating imaging techniques (e.g., echocardiography or Doppler ultrasound) at an early stage may improve detection rates of adult CoA. Future advancements in imaging, such as high-resolution magnetic resonance angiography, may further enhance diagnostic accuracy while minimizing patient risk (10).

Although the surgical outcome in this case was successful, alternative treatment strategies may be appropriate for similar cases. Hybrid approaches combining endovascular techniques with open repair could be effective for patients with complex CoA, reducing surgical trauma while addressing extensive vascular abnormalities. Long-term management is crucial for this patient due to the risks of aneurysm formation, graft occlusion, and recurrent hypertension. Given that untreated descending thoracic aortic aneurysm patients have a 5-year survival rate of approximately 54%, with aortic rupture being the leading cause of death, we recommend implementing a standardized postoperative follow-up protocol. This should include regular imaging studies to assess graft patency and aneurysm status, combined with blood pressure monitoring, to enable early detection of complications and timely intervention. Advances in graft materials and techniques, such as bioengineered vascular grafts, may further improve long-term outcomes by reducing thrombosis risk and enhancing biocompatibility (11).

A major limitation of this report is the duration of follow-up. Six months to one year is not sufficient to assess the long-term patency and durability of the graft, nor to fully evaluate risks such as late graft occlusion, pseudoaneurysm, or recurrence of hypertension. Longer-term follow-up and larger case series are needed to better understand the outcomes of surgical and endovascular repair in adult CoA.

This case underscores the importance of individualized treatment strategies for CoA in adult and elderly patients with complex vascular anatomy. It highlights the role of multidisciplinary collaboration in achieving successful outcomes and emphasizes the need for improved diagnostic protocols to enable early detection and management of CoA in adults.

## Data Availability

The original contributions presented in the study are included in the article/Supplementary Material, further inquiries can be directed to the corresponding author.
